# Progress and current challenges with the quantum similarity model

**DOI:** 10.3389/fpsyg.2015.00205

**Published:** 2015-02-25

**Authors:** Emmanuel M. Pothos, Albert Barque-Duran, James M. Yearsley, Jennifer S. Trueblood, Jerome R. Busemeyer, James A. Hampton

**Affiliations:** ^1^Department of Psychology, City University LondonLondon, UK; ^2^Department of Cognitive Sciences, University of California, IrvineIrvine, CA, USA; ^3^Department of Psychological and Brain Sciences, Indiana UniversityBloomington, IN, USA

**Keywords:** similarity, similarity judgment, quantum probability theory, metric axioms, symmetry, diagnosticity

This opinion paper reviews progress with the quantum similarity model (QSM), which was proposed by Pothos et al. (2013). In the QSM, concepts are associated with subspaces, the mental state is a state vector in a Hilbert space, and similarity between two concepts is computed in terms of the sequential projection, between the corresponding subspaces. If there is a relevant context, this is incorporated as prior projections (e.g., Figure 1).

**Figure 1:**
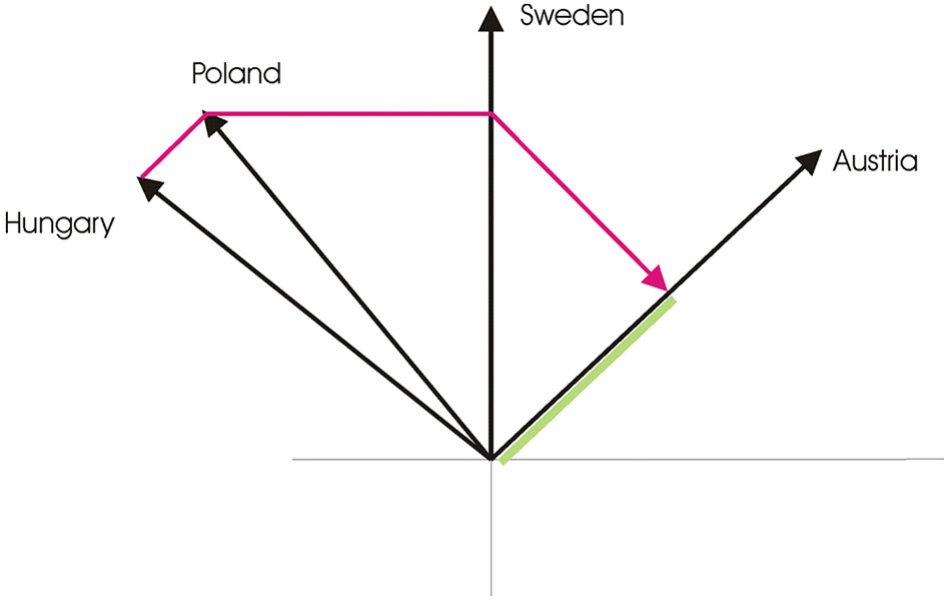
**An illustration of the series of projections, relevant to the similarity of Sweden to Austria, in the context of Poland and Hungary (assuming all countries are represented as rays)**. The red line shows the series of projections: *P _Hungary_P_Poland_P_Sweden_P_Austria_* | ψ 〉. Similarity is the squared length of this projection (indicated in green). The last two projections correspond to the similarity comparison and the first two to the context. Note, also, that projections across context elements need be counterbalanced, but, for simplicity, in the example we illustrate only one order (from Hungary to Poland). Finally, with this context, the similarity judgment is going to be higher, compared to having context elements not grouped together.

The QSM was developed as a way to primarily cover the empirical findings of Tversky (1977). Tversky (1977) reported a series of results for (mostly) simple (non-analogical, see below) pairwise similarity judgments. Tversky's (1977) research severely challenged the dominant similarity models at that time, based on metric spaces and distances. Such models are constrained to obey the metric axioms (as long as similarities are simple functions of distances). Yet, in his seminal work, Tversky reported violations of all three metric axioms (minimality, symmetry, triangle inequality), in the similarity judgments of naïve observers. Moreover, Tversky reported a so-called diagnosticity effect, where the same similarity judgment could change greatly, depending on which other stimuli were present in a (broadly) relevant context set.

All of Tversky's (1977) findings reveal intuitions about human similarity that are, initially at least, very surprising. For example, how can it be possible that the similarity between (simplifying his example) China and Korea be less than Korea and China? Yet, some thought shows that we indeed prefer to judge a non-prominent object (e.g., Korea) as more similar to a prominent one (e.g., China), as compared to the reverse order. Equally, how can it be possible that Austria is seen as more similar to Sweden than to Hungary in the context of Poland, but more similar to Hungary than to Sweden in the context of Norway?

Tversky's findings have been a major focus of subsequent theoretical work on similarity judgments. Some of the most prominent models are the distance-density model (Krumhansl, 1978), general recognition theory (Ashby and Perrin, 1988), and the generalized context model (Nosofsky, 1988; this is a theory of categorization, rather than similarity, yet Nosofsky considered in his influential work how to accommodate Tversky's findings as well, e.g., Nosofsky, 1991). Limited space prevents us from a detailed analysis of this work. Overall, we think that while such work has provided many excellent intuitions regarding human similarity, its application to Tversky's (1977) findings is not uniformly satisfactory. This was a consideration that in part motivated the QSM.

Another motivating consideration has been the recently proposed model for the conjunction fallacy, based on quantum theory (Busemeyer et al., 2011). The conjunction fallacy is a famous result in decision making, whereby naïve observers judge a hypothetical person, Linda, to be more likely to be both a Bank teller and a feminist, than just a bank teller (Tversky and Kahneman, 1983). Of course, such a result is paradoxical, if one employs the rules of classical probability theory. Tversky and Kahneman (1983) suggested that naïve observers in their experiment employed a so-called representativeness heuristic, judging Linda to be more similar to the category of bank tellers and feminists. Thus, at the heart of the explanation for the conjunction fallacy is the idea that participants employ a similarity process (see also Shafir et al., 1990, for further validations of this idea). The quantum model for the conjunction fallacy indeed reflects operations that involve the overlap of a state vector (representing the mental state of participants) and subspaces (which correspond to different concepts in the participants' knowledge space, e.g., the idea that a woman can be a feminist; cf. Sloman, 1993). Thus, we were interested in whether the quantum model for the conjunction fallacy could be extended, more or less as it is, to function as a model of some aspects of similarity. This was indeed the approach that was adopted by Pothos et al. (2013) and the QSM is structurally and procedurally nearly entirely equivalent to Busemeyer et al's (2011) model for the conjunction fallacy. That the same principles can provide a route for explaining both aspects of decision-making and similarity enables the exciting possibility that a formal unification may be possible between these two seemingly disparate aspects of cognition.

One emphasis of the QSM has been the demonstration of asymmetries in similarity judgments. In the QSM this arises in part because concepts are represented as subspaces. Note that the use of subspaces as such is not a uniquely quantum feature of the QSM, but the lack of commutativities in projection sequence (which contributes to the emergence of asymmetries) is. Subspaces can have rich inner structure, corresponding e.g., to the characteristics of a concept. Thus, concepts for which we have more knowledge (such as China, if we imagine ourselves in the shoes of Tversky's participants in 1977) will be represented by a higher dimensionality subspace, contrasting with concepts for which we have less knowledge (such as Korea). Together with an assumption that the mental state prior to a (simple) similarity comparison is neutral between the two concepts to be compared, this enables a natural emergence of asymmetries in human similarity judgments, in the predicted direction. More generally, conceptually, we think that representations as subspaces are an important advance. This is because representations in the QSM can have inner structure, not just in terms of a list of characteristics, but also in terms of how the characteristics relate to each other. By contrast, in traditional spatial representations, with concepts being represented as points or vectors, there is no possibility of such structure at all. This would be the case even in e.g., Latent Semantic Analysis approaches to representation, which have proved extremely useful and influential (e.g., Dumais, 2004; see also Kitsch, 2014; Kitsch, for an insightful comparison between the QSM and Latent Semantic Analysis; note that in Kitsch's (2014), approach, vectors are given variable length, and this can capture differences in degree of knowledge). But even in Tversky's (1977) feature-based approach, concepts would be lists of features, and Tversky (1977) did not consider how dependencies among features could be incorporated in his model.

The way violations of the triangle inequality arise in the QSM is very similar to how Tversky (1977) suggested such effects arise. Because in the QSM representations are subspaces, different regions in the overall space end up reflecting the features characteristic of the corresponding concepts. So, for example, imagine a region in the overall space with Russia and Cuba. This region will overall reflect the property of communism, noting that both Russia and Cuba are consistent with this property (thinking again as participants in Tversky's experiment in 1977). Then, imagine a region different to the first one containing Cuba and Jamaica. The shared characteristic of Cuba and Jamaica is their geographical proximity (they are both in the Caribbean), so this second region will likewise correspond to this property. It should be hopefully straightforward to then see how, if Cuba is on the boundary of the communism and Caribbean regions in psychological space, we can have Cuba highly similar to Russia, Cuba highly similar to Jamaica, but Russia and Jamaica dissimilar from each other, thus violating the triangle inequality. It has to be noted, however, that the triangle inequality is not a challenge for standard (non-linear) distance-based models of similarity. This is because the triangle inequality is already violated if one relates distance and similarity, via a non-linear function (such as the standard exponentially decaying function; Nosofsky, 1984; Shepard, 1987). Nevertheless, it is clearly important for a similarity model to cover violations of the triangle inequality in a convincing manner. Note, violations of the triangle inequality have been the focus of an alternative similarity model, based on quantum theory (Aerts et al., 2011).

A great focus for further work with the QSM concerns the diagnosticity effect. This is because the diagnosticity effect has proved difficult to replicate (e.g., see Evers and Lakens, 2014). We are interested in exploring whether the QSM model can provide insight into why the diagnosticity effect has proved elusive in its replicability. The diagnosticity effect is also significant because the quantum formalism, overall, is often said to embody strong contextual influences. So, perhaps, quantum theory would be particularly suitable for modeling context effects in similarity judgments? Well, the diagnosticity effect does emerge fairly naturally from the QSM, but the mechanisms that allow this are not the traditional contextual mechanisms in quantum theory (e.g., relating to entanglement or incompatibility). In the QSM, the contextual influences relevant to the diagnosticity effect emerge from the way prior projections are used to capture sensitivity to the grouping of context elements. In other words, the difficulty lies in the fact that contextual influences in similarity specifically depend on the degree of grouping of some of the options in the relevant choice set. For example, in Tversky's (1977) demonstration, participants were asked to decide which country is most similar to Austria, between Sweden, Hungary, and Poland. More participants chose Sweden, but when the choice set included Sweden, Hungary, and Norway, they chose Hungary. What we might call the “traditional” mechanisms for contextual influences in quantum theory are not sensitive to the similarity structure of the relevant options.

Contextual influences in the QSM arise in the following way. Similarity computations are based on projecting (laying down) the state vector (which represents the current mental state) onto different subspaces (which represents the concepts relevant in the similarity task; Figure 1). This projection operation can be highly order dependent in quantum theory. Of relevance, the outcome of a projection sequence is sensitive to the grouping of the subspaces across which projection takes place. If the subspaces are grouped together, then a projection sequence preserves the length of the state vector and vice versa. Thus, to account for the diagnosticity effect in the QSM, we postulated that, in a forced choice task (such as the one employed by (Tversky, 1977), in his diagnosticity formulation), prior to the projections corresponding to the elements in the similarity judgment, there would be projections corresponding to the other elements in the choice set. So, for example, if a participant is considering which between Sweden, Hungary, Poland is most similar to Austria, and is specifically evaluating the option of Sweden, then the similarity comparison would consist of projections from Sweden to Austria, but also there would be prior projections to Hungary and Poland. Using this scheme, with fairly minimal assumptions about the representation of the relevant stimuli, the diagnosticity effect emerges from the QSM.

One important challenge in further developing the QSM is further formalizing the way contextual influences are taken into account. The idea of incorporating context as prior projections works well, but it has a heuristic feel to it. Can the QSM be extended such that these prior projections can be motivated in a more rigorous way (cf. Lambert-Mogiliansky et al.'s, 2009, quantum model of framing effects)? Moreover, as noted, can the QSM generate any new predictions regarding the emergence or suppression of the diagnosticity effect? Since Tversky's (1977) work, there has not really been much further examination (or little that has reached the journals), which is surprising (in the sense that the idea of context in similarity judgments seems like a vast topic). These questions are an important focus for our current work with the QSM.

Another important focus concerns so-called analogical similarity judgments (e.g., Goldstone, 1994; Gentner and Markman, 1997). Analogical similarity refers to the idea that, for example, if we are comparing two people, Jim and Jack, if they both have black hair, this will increase their similarity, but if Jim has black hair and Jack has black shoes (and blond hair), this will have less impact on their similarity. That is, work on analogical similarity recognizes that objects often consist of separate components. Commonalities on matching components (e.g., black hair) increase similarity more so than commonalities on mismatching components (e.g., black hair and black shoes). It is currently unclear whether there is a genuine distinction between cognitive processing corresponding to basic similarity tasks (as in Tversky, 1977) and analogical similarity ones (some researchers have suggested that different cognitive systems may mediate the two types of judgments; Casale et al., 2012). Nevertheless, there have been largely separate corresponding literatures, with different objectives. We think that the QSM can be extended to incorporate analogical similarity, because quantum theory already has extensive machinery in place for combining individual components into a whole (cf. Smolensky, 1990). We have been pursuing an approach based on tensor products and we are optimistic that a concrete proposal will be forthcoming soon (Pothos and Trueblood, 2015).

Finally, the QSM is only part of a broader effort within the quantum cognition community to understand similarity using quantum processes. A more challenging, though important objective, would be to examine the formal relation between QSM and, for example, Aerts's (2009) model for conceptual combination or Lambert-Mogiliansky et al.'s (2009) model of framing effects.

## Conflict of interest statement

The authors declare that the research was conducted in the absence of any commercial or financial relationships that could be construed as a potential conflict of interest.
